# Extracellular Vesicles as Drug Targets and Delivery Vehicles for Cancer Therapy

**DOI:** 10.3390/pharmaceutics14122822

**Published:** 2022-12-16

**Authors:** Sai V. Chitti, Christina Nedeva, Raja Manickam, Pamali Fonseka, Suresh Mathivanan

**Affiliations:** 1 Department of Biochemistry, La Trobe Institute for Molecular Science, La Trobe University, Melbourne, VIC 3086, Australia; 2 Department of Biomedical Engineering, The Kavery Engineering College, Mecheri, Salem 636453, Tamil Nadu, India

**Keywords:** extracellular vesicles, cancer therapy, drug targets, drug delivery vehicles

## Abstract

Extracellular vesicles (EVs) are particles that are released from cells into the extracellular space both under pathological and normal conditions. It is now well established that cancer cells secrete more EVs compared to non-cancerous cells and that, captivatingly, several proteins that are involved in EV biogenesis and secretion are upregulated in various tumours. Recent studies have revealed that EVs facilitate the interaction between cancer cells and their microenvironment and play a substantial role in the growth of tumours. As EVs are involved in several aspects of cancer progression including angiogenesis, organotropism, pre-metastatic niche formation, fostering of metastasis, and chemoresistance, inhibiting the release of EVs from cancer and the surrounding tumour microenvironment cells has been proposed as an ideal strategy to treat cancer and associated paraneoplastic syndromes. Lately, EVs have shown immense benefits in preclinical settings as a novel drug delivery vehicle. This review provides a brief overview of the role of EVs in various hallmarks of cancer, focusing on (i) strategies to treat cancer by therapeutically targeting the release of tumour-derived EVs and (ii) EVs as valuable drug delivery vehicles. Furthermore, we also outline the drawbacks of the existing anti-cancer treatments and the future prospective of EV-based therapeutics.

## 1. Introduction

The International Society for Extracellular Vesicles (ISEV) proposed the term “extracellular vesicle” as a generic nomenclature for cell-released vesicles [1]. EVs are broadly classified into various subtypes based on operational terms such as (a) size: small EVs (i.e., exomeres < 50 nm (nanoparticles), exosomes 30–200 nm, small ectosomes 30–200 nm) and large EVs (i.e., ectosomes 100–1000 nm, migrasomes 500–3000 nm, apoptotic bodies 1000–5000 nm, large oncosomes 1000–10,000 nm); (b) biochemical composition (i.e., CD63+, CD81+, CD9+, annexin A5-stained EVs); and (c) descriptions of the conditions or cell of origin (i.e., hypoxic EVs, podocyte EVs) [2]. Small EVs (sEVs), except the membrane-less exomeres, are thought to mostly originate as intraluminal vesicles formed by inward invagination of the endosomal membrane and released into the extracellular space upon fusion of the multivesicular bodies (MVBs) with the cell surface [3,4]. In addition, outward budding of the plasma membrane also results in the release of sEVs [2]. Large EVs (lEVs) such as ectosomes are formed by direct outward budding or protrusion from the plasma membrane [5]. Apoptotic bodies are formed by outward blebbing of the plasma membrane of cells undergoing apoptosis [6]. Several seminal studies suggested that within these EV populations, various subpopulations may exist and isolation of one specific subtype of EVs has proven to be a challenging task in EV purification [7]. Similar to their heterogeneity in size, the content of EVs is also highly variable, which likely reflects the dynamic state of the cell [7]. The functional proteins, lipids, messenger RNAs (mRNAs), micro RNAs (miRNAs), noncoding RNA (ncRNA), genomic DNA, and surface molecules within the EVs vary according to cell type and the precise pathophysiological status of the cells [8]. EVs, once merely described as extracellular debris, are now considered to be critical mediators in the regulation of numerous physiological processes such as embryonic development [9], maintenance of tissue homeostasis [10], and immune regulation [11], and pathological processes such as cardiovascular, autoimmune, and neoplastic diseases [12,13]. Importantly, EV cargo can also be used as biomarkers and therapeutic tools for a variety of diseases such as Parkinson’s, cardiovascular, and cancer [14,15,16,17]. In this review, we provide an overview of the current knowledge on the role of EVs in cancer progression and discuss strategies to block the release of cancer cell EVs as an avenue for cancer therapy. Furthermore, we discuss current challenges with the existing anti-cancer therapies and the advantages of EVs as drug delivery vehicles.

### 1.1. The Dynamic Role of Tumour-Derived EVs in the Pathogenesis of Cancer

**Figure 1:**
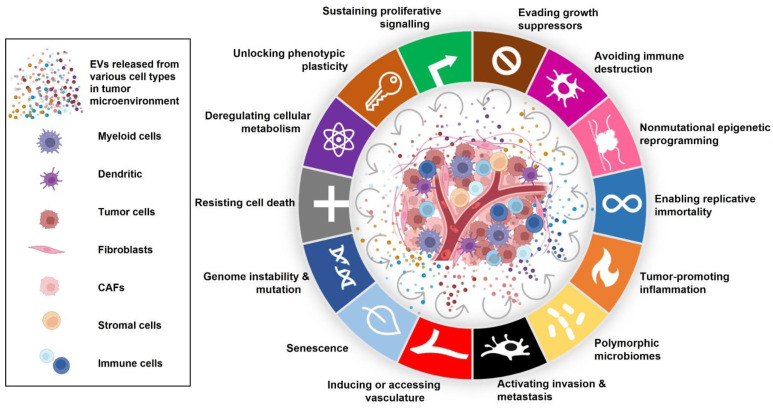
Role of EVs in the hallmarks of cancer. EVs secreted from various cell types in the tumour microenvironment can modulate the hallmarks of cancer which then leads to cancer progression and a poor prognosis.

EVs have been associated with almost every hallmark and paraneoplastic feature of cancer [18,19,20]. With the ongoing research on tumour development and progression, EVs have added a layer to the previously unanswered questions related to understanding the complexity of the disease. Recent studies have revealed that EVs facilitate the interaction between cancer cells and their microenvironment and play a substantial role in the growth of tumours [21,22,23]. EVs derived from cancer cells have been shown to actively contribute to almost all the steps necessary for the progress of carcinomas by transforming neighbouring cells within the tumour microenvironment, promoting invasion [24], pre-metastatic niche formation and metastasis, organotropism [25], angiogenesis [26], immune evasion [27,28], chemoresistance [29,30], and cancer-associated muscle [31,32] and fat loss [33,34] (Figure 1). 

### 1.2. EVs Promote Cancer Progression and Angiogenesis

It has been proposed that EVs contribute to the non-cell-autonomous mechanism of tumour initiation and progression [35,36]. In 2008, two independent studies reported that tumour-derived EVs can influence the behaviour of cancer cells, as well as normal cells by horizontal transfer of genetic information [37] and oncogenic protein [38]. Later on, breast cancer cell-derived EVs containing miRNAs were implicated in inducing the proliferation and transformation of non-cancerous cells into tumour-forming cells [22]. Similarly, Stefanius and colleagues demonstrated the potential of pancreatic cancer cell-derived EVs in the initiation of transformation to malignant cells by inducing random mutations in the recipient cells [21]. Our laboratory showed that EVs derived from human colorectal cancer cells (LIM1215) carrying mutant β-catenin could alter the Wnt signalling pathway in the recipient cells bearing wild-type β-catenin [23]. Furthermore, intravenous administration of LIM1215 EVs increased the tumour burden of mice implanted with cancer cells [23]. In addition to the initiation and promotion of tumours, several studies have reported on the proangiogenic potential of tumour-derived EVs [39,40,41,42,43]. Upon internalization into endothelial cells, EVs bioactive compounds such as miRNA, ncRNA, and proteins that have the potential to initiate an angiogenesis switch targeting various mechanisms such as vascular endothelial growth factor (VEGF)/VEGF receptor, WNT, Notch, and hypoxia-inducing factor, thus contributing to tumour angiogenesis [41,44]. Furthermore, tumour-derived EVs have been shown to inhibit antitumour responses and thereby promote angiogenesis and tumour progression [45,46].

### 1.3. Role of EVs in Senescence and Evading Apoptosis 

Senescence is a state of proliferative arrest that prevents neoplastic events [47]. However, accumulating evidence suggests dual roles of the senescence-associated secretory phenotype (SASP) largely driven by EVs [48,49,50,51]. Lehmann et al. reported that irradiated prostate cancer cells undergoing senescence released more EVs in the p53-dependent mechanism compared to the untreated controls [52]. Similarly, Takasugi et al. not only demonstrated the increased secretion of sEVs from cells undergoing cellular senescence but also reported the growth-promoting effect of those sEVs via EphA2/ephrin-A1 reverse signalling [48]. Furthermore, Han and colleagues revealed that senescent stromal cells promote cancer resistance via excessive release of sEVs [53]. EVs have also been shown to promote anti-apoptotic activity in cancer cells [54,55]. For instance, Pavlyukov et al. demonstrated that glioblastoma cells shed apoptotic EVs containing splicing factor RNA-binding motif 11 which switches splicing of MDM4 and cyclin D1 towards the expression of more oncogenic isoforms in the recipient cells, thereby impairing apoptosis [54]. Yang et al. investigated the role of bladder cancer-derived EVs in evading apoptosis and reported that the inhibition of apoptosis is due to the upregulation of Bcl-2 and cyclin D1 levels and the downregulation of Bax and caspase 3 [56]. 

### 1.4. Organotropism, Pre-Metastatic Niche Formation, and Fostering of Metastasis by EVs

One of the critical phases of tumour progression is the establishment of the pre-metastatic niche leading to metastasis. Secretory factors released from the tumour cells have been shown to induce vascular leakiness and recruit pro-angiogenic immune cells and promote organotropism [57,58,59,60,61]. In this context, EVs have been implicated in pre-metastatic niche formation (PMF) and fostering metastasis by several seminal studies [62,63,64]. Furthermore, tumour-derived EVs containing specific cargo have been shown to localise to specific organs and hence aid in organotropism [25]. Various biomolecules that were enriched in the tumour-derived EVs have been implicated in PMF and metastasis in multiple cancers including cMET [62], CD151, Tspan8 [65], CD97 [66], CXCR4 [67], MIF [63], EGFR [68], integrins [25], CEMIP [69], miR-181c [70], miR-105 [58], miR-21 [71], and MMP1 mRNA [72]. Hoshino and colleagues recently demonstrated the correlation between EV integrins expression patterns and specific tissue organotropism [25]. EVs positive for integrins α_6_β_4_ and α_6_β_1_ directed lung tropism, whereas α_v_β_5_ integrins were driving tropism to the liver [25]. Collectively, several recent studies articulate the contribution of specific cargo within the EVs in driving organotropism, PMF, and metastasis.

### 1.5. Interference of EVs with Anti-Cancer Therapies and Evasion of Immune Surveillance 

It has been proposed that one of the mechanisms by which cancer cells become resistant to anti-cancer therapies is via the release of EVs [73,74]. EVs have been shown to influence chemoresistance through the transfer of vesicle cargo (from drug-resistant donor cells to drug-sensitive recipient cells) and the sequestering of chemotherapeutic drugs [75]. Ma et al. showed that transient receptor potential channel 5 (TrpC5) containing EVs transferred chemoresistance to the non-chemo-resistant cells by stimulating multidrug efflux transporter P-glycoprotein production [76]. Jorfi et al. demonstrated the ability to inhibit EVs as a way to reduce anti-cancer drug resistance using the prostate cancer cell line PC3 [77]. Similarly, the role of EVs in immune evasion has also been examined. For instance, several seminal studies have reported on the role of tumour-derived EVs as decoys or shields in protecting the target cells from antibody detection [27,28]. Aung and colleagues showed that B-cell lymphoma-derived EVs bearing CD20 act as a “trap” by binding therapeutic anti-CD20 antibodies, in turn protecting the cancer cells against the immune system [27]. 

### 1.6. EV Associated Cargo Promotes Cancer-Induced Muscle Atrophy and Lipolysis

During the advanced stage of cancer, many patients notably experience a significant reduction in their body weight due to progressive multi-organ wasting. This wasting syndrome is known as cancer cachexia [78]. In this context, studies have highlighted the role of tumour-secreted EVs in promoting muscle catabolism [79]. A study by Zhang et al. reported the ability of tumour-derived EVs, containing heat shock proteins 70 and 90 (Hsp70/90), to induce muscle wasting [31]. In 2021, Gao et al. showed that EVs containing prolyl 4-hydroxylase subunit beta (P4HB) mediate muscle wasting during oesophageal squamous cell carcinoma [32]. Hsp70/90 and P4HB induce the activation of the ubiquitin-proteasome pathway and apoptosis, respectively [31,32]. One of the key features of cancer-associated wasting is evident in adipose tissue loss, which is known as lipolysis [33,80]. Emerging evidence suggests that tumour-derived EVs can also induce lipolysis [33,81]. For instance, Sagar et al. have shown that pancreatic cancer-derived EVs, containing adrenomedullin, promote lipolysis in the subcutaneous adipose tissue via p38-ERK1/2 and phosphorylation of hormone-sensitive lipase [33]. In 2021, Hu and colleagues demonstrated that the lipolysis and browning associated with Lewis lung carcinoma (LLC) tumour are regulated by cancer cell-derived EVs containing parathyroid hormone-related protein [34].

## 2. Blocking EV Biogenesis, Trafficking, Release, and Recipient Cell Uptake as a New Cancer Therapeutic Avenue

**Figure 2:**
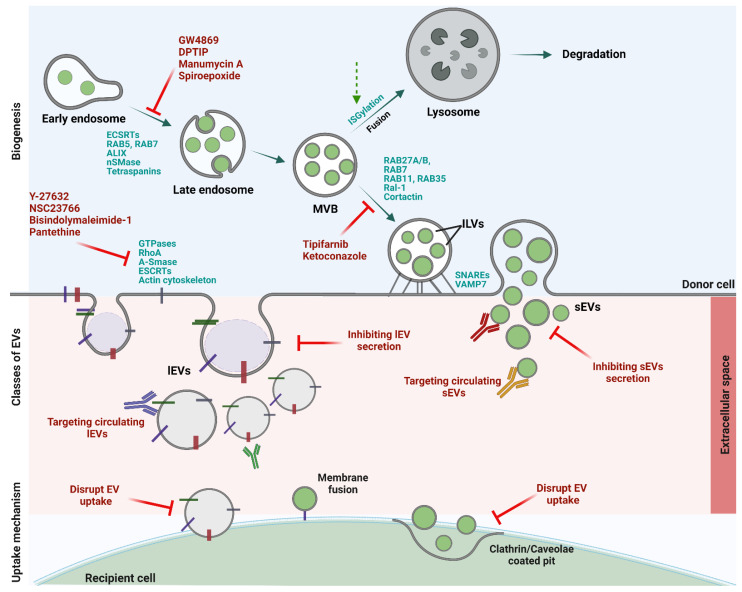
Therapeutic targets and agents against cancer-derived EVs. Various classes of EVs are generated through different biogenesis pathways and involve several proteins. These proteins can be therapeutically targeted to inhibit their biogenesis, secretion, and uptake by the recipient cells. Red lines: pharmacological agents affecting biogenesis, secretion, and uptake of EVs. Green dotted line: activator/s of ISGylation that promote fusion of MVBs with lysosomes for degradation.

Given the evidence involving tumour-derived EVs in all the steps of cancer progression, it can be speculated that targeting EV biogenesis and secretion, activation of MVBs fusion with lysosomes and subsequent degradation, capturing of circulating tumour-derived EVs, and inhibiting the uptake of EVs by the recipient cells might be a promising approach to treat cancer recurrence and associated paraneoplastic syndromes (Figure 2) [82,83]. However, several issues relating to the safety and efficacy of this approach need to be examined in pre-clinical models. Several studies have proposed that targeting tumour-derived EVs by disrupting the proteins involved in EV biogenesis and secretion may be a new treatment option for cancer [57,62,84,85]. From the initial step of EV formation to their secretion into the extracellular environment, the coordinated effort of several protein networks is needed [86]. Although most of the sEVs and lEVs generation occurs at distinct sites, the two different-sized EVs share common intracellular mechanisms and sorting machinery involved in their biogenesis [86]. The release of sEVs from tumour cells can be inhibited by blocking the proteins involved in their biogenesis such as endosomal sorting complexes required for transport proteins (ESCRT-dependent), tetraspanin family proteins, and neutral sphingomyelinases (ESCRT-independent) [87]. Alternatively, blocking the sEVs trafficking and secretion by targeting Rab family proteins, soluble N-ethylmaleimide-sensitive factor attachment protein receptors (SNAREs), Rac-1, and actin cytoskeletal proteins have also been proposed [64,84,88]. LEVs formation and release can potentially be blocked by targeting small GTPase RhoA [89], membrane cholesterol [90], and Ca^2+^-dependent enzymatic machinery that aids in the externalization of phosphatidylserine (PS) [91]. Hence, for the inhibition of tumour-derived EVs, many pharmacological agents were explored. These include compounds that target the shedding of lEVs (Bisindolylmaleimide-1, pantethine, Y-27632, NSC23766), sEVs (GW4869, imatinib, manumycin A, spiroepoxide, DPTIP, ketoconazole, tipifarnib), or both. Cannabidiol, a phytocannabinoid, has been shown to block the release of both sEVs and lEVs by decreasing CD63 expression in prostate, hepatocellular, and breast cancer cells [92]. Many drugs are used in the literature to block EV biogenesis and/or secretion and it is not clear whether they inhibit both lEVs and sEVs. 

### 2.1. Pharmacological Agents That Affect lEVs Formation and Secretion

Y-27632: Y-27632 is a selective, highly potent, reversible, competitive inhibitor of p160-Rho-associated coiled coil-protein serine/threonine kinases 1 and 2 (ROCK1 and ROCK2) [93]. Y-27632 inhibits kinase activity, and the inhibition is reversible by ATP in a competitive manner [94]. ROCK1 and ROCK2 have been known to regulate actin cytoskeletal remodelling and actomyosin contraction via the activation of adducin, which maintains actin-network assembly; the ezrin, radixin, and moesin proteins (ERM), which is important for actin–membrane linkage; LIM-kinase (LIMK), which inactivates cofilin that regulates actin filament stabilization and the branching of actin filaments; and myosin light chain (MLC) [95]. Li and colleagues investigated the role of ROCK1 and ROCK2 in EV formation in triple-negative breast cancer (MDA MB 231), glioblastoma (U-87), and ovarian cancer (HeLa) cells [89]. Treatment of MDA MB 231, U-87, and EGF-stimulated HeLa cells with the dominant-active form of Rho-GTPase increased the amount of EVs in the conditioned medium, whereas knockdown of RhoA inhibited the release of EVs from the cancer cells. Interestingly, treatment with Y-27632 eliminated the presence of EVs along their membrane, confirming the role of Rho-GTPases in the formation of EVs [89]. Similarly, the role of Rho-GTPases in EV formation and the potential utility of Y-27632 as an inhibitor for EV formation was documented in other disease models [96,97]. 

Bisindolylmaleimide-1 (BIM-1): BIM-1 is a highly selective, reversible inhibitor targeting the ATP-binding site of various isoforms (α-, βI-, βII-, γ-, δ-, and ε) of protein kinase C [98]. The mechanism driving the release of lEVs is mainly regulated by calcium and the externalization of phosphatidylserine (PS) [95]. Stratton and colleagues demonstrated a 75% inhibition of EV release, independent of intracellular calcium levels, in the PC3 cell lines upon treatment with BIM-1 [99]. Kosgodage and co-workers reported that breast and prostate cancer cells treated with BIM-1 significantly inhibited EV release without inducing apoptosis of cancer cells [91]. It has been proposed that the mechanism by which BIM-1 reduces the levels of EV secretion is via decreased externalization of PS [91,99]. 

Pantethine: Pantethine is a derivate of vitamin B5-pantothenic acid and is approved for use in the clinic to decrease the levels of plasma triglycerides and increase the levels of high-density lipoproteins. The mode of action of pantethine is by decreasing the total level of cholesterol and fatty acid synthesis [100]. Roseblade et al. showed that pre-treatment of the breast cancer cell line MCF-7 with panthethine reduced the number of EVs released by 24% compared to the control [101]. The reduction in the total number of EVs released might either be due to the decrease in cholesterol in the cell, which mainly regulates the fluidity during membrane lipid re-organization, or the inhibition of the translocation of PS. Additionally, a similar reduction in EV release upon pantethine treatment was reported in other disease models such as systemic sclerosis [102] and cerebral malaria [103]. Other compounds such as calpeptin, U0126, and clopidogrel were also reported to decrease EV formation and release [95]. 

### 2.2. Pharmacological Agents That Affect sEVs Biogenesis and Secretion

GW4869: Since its initial observation in 2008, a membrane-neutral sphingomyelinase (nSMase) inhibitor, GW4869, has been a widely used pharmacological agent for the blocking of ceramide-mediated EV biogenesis [87]. GW4869 is a potent and non-competitive inhibitor that specifically blocks ceramide-mediated inward budding of MVBs and the subsequent release of ILVs as EVs [104]. Cao et al. demonstrated that the acquired cisplatin resistance in ovarian cancer cells is mediated by the increased expression of DNA methyltransferase 1 in EVs [105]. When ovarian cancer cells were treated with GW4869, sensitivity to chemotherapy was restored in resistant cells by decreasing the release of EVs [105]. Matsumoto and colleagues showed that melanoma cancer cell-derived EVs promoted the proliferation of cancer cells by the release and uptake of their EVs [106]. Inhibition of EV release with GW4869 treatment significantly decreased tumour progression both in vitro and in vivo [106]. Similarly, in our study, we reported the decreased release of sEVs from breast cancer cells upon treatment with GW4869 [107].

Manumycin A, spiroepoxide, and DPTIP: These drugs have been shown to selectively block the release of sEVs from various cancer cells by targeting neutral sphingomyelinases or ESCRT machinery [108,109,110]. Datta et al. employed a high throughput screen and identified drugs that specifically target EV biogenesis and secretion [108]. Manumycin A (MA), a natural microbial metabolite, significantly reduced EV number specifically from prostate cancer (C4-2B) cells but not from normal (RWPE-1) cells. ESCRT-0 (Hrs), ALIX, and Rab27a levels were reduced upon treatment with MA; however, MA has not progressed into clinical trials due to associated side effects [108]. Similarly, spiroepoxide has been shown to reduce EV secretion from cultured macrophages by specifically inhibiting nSMase [111]. Rojas and colleagues identified 2,6-Dimethoxy-4-(5-Phenyl-4-Thiophen-2-yl-1H-Imidazol-2-yl)-Phenol (DPTIP), with nanomolar potency, as the most potent, highly selective, and brain penetrable nSMase inhibitor [109]. DPTIP has been shown to inhibit EV secretion from astrocytes by specifically inhibiting nSMase in a dose-dependent manner [109]. However, the inhibitory effect of DPTIP on sEVs released from cancer cells and tumour in vivo models is yet to be examined. 

Tipifarnib and ketoconazole: Datta et al. screened 4580 compounds via quantitative high throughput screening and identified tipifarnib and ketoconazole as key compounds that modulate EV biogenesis and/or secretion from prostate cancer cells [112]. Tipifarnib and ketoconazole are proposed to inhibit EV biogenesis and secretion via both ESCRT-dependent and ESCRT-independent pathways. In addition, these two compounds have been shown to significantly decrease the expression of various proteins involved in EV biogenesis and secretion such as ALIX, nSMase2, and Rab27a in prostate cancer cells but not in normal cells (RWPE-1) [112]. 

### 2.3. The Challenges Associated with EV-Targeted Therapies

Although studies have shown an effective reduction in tumour EV biogenesis and secretion with the use of chemical inhibitors, antibodies, and genetic engineering techniques, such therapies are yet to be approved by the Food and Drug Administration (FDA) [112,113]. One of the major challenges in EV-based drug development is the identification of molecules that have no adverse effects on cellular homeostasis [112]. Current consensus suggests that EV-based drug development research needs to shift focus to developing strategies that block EV secretion from cancer cells, as opposed to targeting EV biogenesis, since it involves multiple pathways (ESCRT-dependent, ESCRT-independent, and tetraspanins) and due to the inconsistency in the expression of various ESCRT-dependent and ESCRT-independent proteins across various cancer types. For example, depletion and overexpression studies show that Rab27 is the only GTPase identified to entirely demonstrate a change in the number of EVs released. However, Rab27 expression is inconsistent across cancer cell lines [114]. HeLa cells require Rab27a and Rab27b, whereas 4T1 and TS/A breast cancer cells require Rab27a [114]. This discrepancy across various cancers does not make Rab proteins an ideal candidate for blocking EV biogenesis from a clinical point of view. One major challenge is to find therapeutic approaches that interfere with these pathways with sufficient specificity in tumour cells without affecting normal cell function. Another critical issue that must be considered is that blocking one specific type of EV may result in the increased secretion of the other type/s of EVs, meaning that blocking sEVs release might result in the increased secretion of IEVs and may lead to pathophysiological changes. Additionally, most of the above-mentioned experimental evidence of EV-targeted therapies was obtained in vitro and hence pre-clinical studies are required to confirm the safety and efficacy of the proposed therapies.

## 3. Extracellular Vesicles as Drug Delivery Vehicles

The effective delivery of drugs and molecules for disease therapy has been met with many challenges over the years. This is mainly due to limitations of free drug/molecule administration including a lack of stability in the body, poor bioavailability, tissue absorption issues with specificity, and undesirable adverse effects [113]. To overcome these issues, nanotechnology has been exploited to improve the efficacy of drug delivery. Since their development in 1965, lipid particles (LNPs) have gained attention as nano-drug carriers due to their size and ability to traffic small molecules [115]. In the 1990s, LNPs were FDA approved for encapsulating small molecules such as doxorubicin and daunorubicin for cancer treatment [116,117]. Within the last decade, further advances have been made with clinical trials utilising LNP–mRNA vaccine formulations for cancer immunotherapy and against SARS-CoV-2, responsible for the recent COVID-19 pandemic (NCT02316457) [118]. A study by Alanazi et. al has shown that 5-fluorouracil-loaded lipid particles demonstrate a higher in vitro cytotoxicity compared to free drug alone when treated on hepatocellular carcinoma (HCC) cells [119]. Despite multiple benefits attributed to the application of liposomes as drug delivery systems, there are many disadvantages associated with their application. Some limitations associated with LNPs include high production costs and comparatively reduced drug loading efficiency due to their structure [120,121]. Due to these LNP-associated drawbacks, the appropriate drug delivery is often hindered, and undesirable side effects can occur, such as drug expulsion [121].

In order to overcome LNP drug delivery problems, EVs have recently been considered as the next-generation drug delivery platform [122,123]. EVs are known to possess multiple advantageous characteristics that deem them ideal drug delivery vehicles. One of the major characteristics of EVs is their ability to cross biological barriers, such as the blood–brain barrier, efficiently whilst keeping their structure intact [20]. Another advantage of using EVs as drug vehicles is their ability to reduce the cytotoxicity of drugs. For instance, a study performed by Schindler et al. discussed that free doxorubicin accumulates in the heart causing cytotoxicity. However, EVs loaded with doxorubicin did not accumulate in the heart and thereby limited cardiac side effects [124]. More importantly, unlike current immunotherapy-based techniques which have longer risk/recovery periods, EVs are non-replicative and non-mutagenic and hence have fewer regulatory and adverse effects [125,126].

For the purpose of drug delivery, EVs have been isolated from various cell types including cancer, mesenchymal stem (MSC), immune, and embryonic kidney cells [127]. However, it should be of note that EVs mirror their originating cell and often contain similar content and surface proteins which can affect their function [30]. Moreover, depending on their origin and route of administration, the biodistribution of EVs may change [128,129]. For instance, Wicklander et al. have shown that EVs from various mouse cell types home in varying locations with different percentages in vivo [128]. Certain cell types are also known to secrete more EVs than other cell types [130]. Hence, it is crucial to select the cell type that is best suited to isolate EVs for drug delivery studies. 

**Figure 3:**
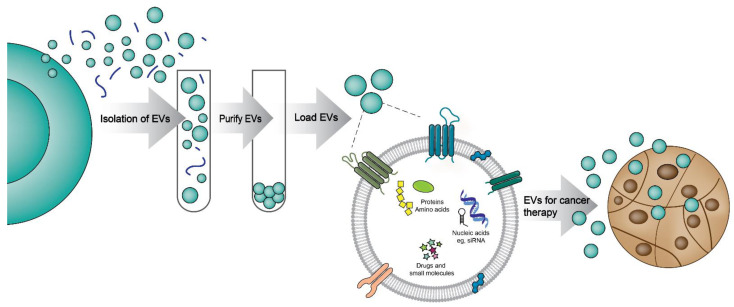
EVs are diverse nanoparticles that can be packaged with cargo and delivered as cancer therapy. EVs can be isolated from various sources including cells, serum, and milk. They can be effectively purified and loaded with different molecules and small drugs for effective delivery to cancer cells.

MSCs are widely used for EV production (MSC–EVs) as they possess unique tissue regeneration [131,132,133] and immune modulation [134] properties. Hence, there are several studies that have used MSC–EVs as drug delivery systems. Pascucci et al. were able to demonstrate that MSC–EVs containing paclitaxel can inhibit the proliferation of pancreatic cancer cells [135]. Whereas Wei et al. showed that doxorubicin-containing EVs enhance the cellular uptake and decrease the growth of osteosarcoma cells with reduced cytotoxicity compared to free doxorubicin [136]. Similar to MSCs, another cell type widely used for EV isolation is the immune cells [137]. EVs secreted from macrophages are used to treat various cancer types such as pancreatic, lung, and breast cancer [130,137,138]. These studies showed that drug-loaded EVs co-localise with cancer cells and exert a potent anti-cancer effect in mouse models compared to free drugs (Figure 3). Dendritic cell-derived EVs are also used to treat breast cancer in pre-clinical models. Tian et al. demonstrated that EVs carrying doxorubicin can cause growth inhibition of breast cancer cells both in vitro and in vivo. Doxorubicin-loaded EVs isolated from HEK293 cells were taken up by the cells rapidly, and the doxorubicin was re-distributed within the cell into the nucleus enhancing the potency of the drug [124]. Interestingly, EVs derived from tumour cells have also been employed as drug carriers. EVs derived from pancreatic cancer cells were used to treat pancreatic cancer after loading with either doxorubicin or gemcitabine. Pancreatic cell-derived EVs loaded with gemcitabine were concentrated in the tumour site in vivo and significantly prolonged the survival rates of mice by suppressing the tumour growth with minimal damage to normal tissues. This observation can be attributed to the potential tropism of EVs to the tumour microenvironment, deeming EVs to be competitive drug delivery vehicles for targeted chemotherapy [139].

More recently, studies have shown that bovine milk-derived EVs can be an excellent scalable candidate for use as drug carriers [140]. Unlike cell-derived EVs, a higher number of EVs can be isolated from bovine milk [141]. The systemic administration of milk EVs into mouse models has shown no cytotoxicity or anaphylaxis [142]. More importantly, milk EVs have shown to be highly stable in the gut with low immunogenicity, deeming them promising carriers of chemotherapeutic agents [140,143,144,145]. Furthermore, studies have shown that bovine milk EVs loaded with a range of drugs such as withaferin A, doxorubicin, anthocyanidins, curcumin, docetaxel, and paclitaxel have significantly improved the bioavailability and efficacy of the drug compared to free drugs in both in vitro and in vivo cancer models [140,146,147,148,149]. Hence, it has been proposed that drug-loaded milk EVs are biocompatible, safe, and effective in tumour targeting and a cost-effective mode of cancer treatment. 

After selecting the appropriate cell type to extract EVs, an isolation method needs to be taken into consideration. Commonly used EV isolation techniques include ultracentrifugation, density gradient centrifugation, polymer-based precipitation, size exclusion chromatography, and immunoaffinity pull-down [150,151]. Each technique can be performed alone or in combination to isolate EVs. Each technique or combination of techniques will vary in yield of EVs, depletion of protein contaminants such as albumins and globulins, depletion of lipoproteins, time taken for each isolation, labour intensity, sterility, and the cost of the procedure [150,152]. 

Following the isolation of EVs, an effective method of loading drugs into EVs is required to be selected. Currently, there are multiple methods of loading drugs into EVs (Table 1). Several studies have added chemotherapeutic drugs to the culture medium to be internalised by cells before the isolation of EVs [139,153,154]. This passive loading of drugs into EVs has been achieved by incubating drugs such as doxorubicin with different cell lines prior to the isolation of EVs and in turn, loaded EVs were able to effectively induce apoptosis of treated cancer cells [130]. In a similar study, Ye et al. used methotrexate-loaded EVs to treat glioblastoma. Here, the authors showed that methotrexate-loaded EVs effectively crossed the blood–brain barrier, thereby successfully delivering the drug which translated into increased survival of mice bearing glioblastoma [155]. Another mode of passive loading of EVs is achieved by incubating EVs in situ with chemotherapeutic drugs such as doxorubicin, paclitaxel, and curcumin. This method is effective as it exploits the hydrophobic nature of these drugs [138,156,157,158,159,160].

Alternative to passive methods, active methods of EV drug loading include sonication [138], electroporation [154] or cycles of freeze–thawing [161]. Electroporation creates temporary pores in the membrane of EVs which allows chemotherapeutic drugs to diffuse into the EVs. However, the electroporation method is shown to have limitations such as fusion and aggregation of the vesicles [127]. Nevertheless, a study by Lennaard et al. has shown that altering the loading parameters for electroporation, such as particle number, EV to drug ratio, buffers, the field of strength, and pulse capacitance can improve the EV recovery and increase the drug potency by 190-fold compared to the naked drug alone [162]. In another study, EVs loaded with drugs using the freeze–thaw cycles method has shown to decrease the cytotoxicity of drugs, enhance cancer cell apoptosis, and efficiently deliver drugs to the tumours in vivo [161]. It is unclear at this stage as to whether the freeze–thaw method has any advantage over other methods of drug loading. Sonication is a much harsher method of drug loading as this method requires mechanical energy to work and it runs the risk of altering the biophysical properties of EVs. However, Kim et al. have shown that sonication resulted in a high loading efficacy and drug release compared to incubation and electroporation [138]. Similar observations were also reported by Salarpour and colleagues with encapsulated paclitaxel in EVs, using incubation and sonication methods to treat glioblastoma [154]. Extrusion and detergents such as saponin can also be employed to incorporate drugs into EVs [127]. Saponin is a surfactant that can cause membrane disruptions that generates pores leading to increased membrane permeabilization. One of the limitations of using this compound is the growing concerns about in vivo hemolytic activity that saponin could cause [163]. 

**Table 1:** Different strategies of drug loading into EVs for cancer therapy.

Method	Drugs	Source of EVs	Application	Ref.
Passive loading (Incubation of drug with cells)	Doxorubicin	THP-1 macrophages	Ovarian and prostate cancer therapy	[164]
	Doxorubicin	Pancreatic cancer cells, pancreatic stellate cells, and macrophages	Pancreatic cancer treatment	[130]
	Methotrexate and cisplatin	A2780 human ovarian cancer cell	Hepatocarcinoma and ovarian cancer treatment	[165]
	Methotrexate	L929 cells	Glioblastoma treatment	[155]
	Paclitaxel	Murine SR4987 cells	Ductal pancreatic adenocarcinoma therapy	[135]
	Paclitaxel, Doxorubicin and Gemcitabine	GinPa-MSCs	Oral squamous cell carcinoma therapy	[153]
	Paclitaxel	Bone marrow mesenchymal stromal cells (BM-MSCs)	Myeloma therapy	[166]
	Paclitaxel	Canine mesenchymal stromal cells (cMSCs)	Glioblastoma treatment	[167]
	Doxorubicin	MCF-7 cells	Breast cancer therapy	[168]
Passive loading (Incubation of drugs with EVs)	Paclitaxel	RAW 264.7 macrophages	Lewis lung carcinoma therapy	[138]
	Withaferin A, anthocyanidins, curcumin, paclitaxel and docetaxel	Bovine milk	Lung cancer therapy	[140]
	Paclitaxel	Bovine milk	Lung cancer therapy	[147]
	Celastrol	Bovine milk	Lung cancer therapy	[169]
	Paclitaxel	LNCaP and PC-3 PCa cell lines	Prostate cancer treatment	[160]
	Paclitaxel and doxorubicin	Brain endothelial bEND.3 cells	Brain cancer therapy	[170]
	Aspirin	HT-29 and MDA-MB-231 cells	Metastatic breast and colorectal cancer therapy	[161]
	Paclitaxel and doxorubicin	RAW 264.7 macrophages	Triple negative breast cancer therapy	[137]
	Paclitaxel	U-87 cells	Glioblastoma therapy	[154]
	Gemcitabine	Panc-1 cells	Pancreatic cancer therapy	[139]
	Doxorubicin	MSC	Osteosarcoma therapy	[136]
Active loading (Electroporation)	Doxorubicin	Mouse immature dendritic cells (imDCs)	Breast cancer therapy	[171]
	Doxorubicin	HEK293 cell	Breast cancer therapy	[124]
	Doxorubicin	MDA-MB-231 cells	Cervical cancer therapy	[172]
	Gemcitabine	M1 Macrophages	Chemoresistant pancreatic cancer treatment	[139]
Active loading (Sonication)	Paclitaxel and doxorubicin	RAW 264.7 macrophages	Triple negative breast cancer therapy	[137]
	Gemcitabine	Panc-1 cells	Pancreatic cancer therapy	[139]
	Paclitaxel	U-87 cells	Glioblastoma therapy	[154]
	Triptolide	SKOV3 cells	Ovarian cancer therapy	[173]

### Challenges in the Using EVs as Drug Delivery Vehicles

Even though many studies provided evidence that EVs carrying drugs is an ideal strategy to treat many cancer types, several hurdles need to be overcome [174]. EVs are highly heterogenous in nature, and it is proposed that specific subtypes have different functions based on the cargo [175]. Hence it is important to be cautious when selecting the subtype of EVs that need to be employed [176]. Moreover, EVs reflect the cell of origin and may carry oncogenic cargo that may be counterintuitive in cancer therapy [177]. Furthermore, depending on the cell of origin, the biodistribution and the half-life of EVs may vary drastically [23]. Hence, as the method of biogenesis and the biophysical properties of EVs may vary, it is crucial to obtain a complete preclinical evaluation of cellular, tissue, and animal models before further consideration. Another aspect that needs to be taken into consideration is that EVs need to be free of contaminations from pathogens such as bacteria, viruses, and other EVs from the media such as fetal bovine serum EVs [174]. 

In order to employ EVs for the treatment of cancer or any other disease, upscaling of EV generation and harvesting needs to be further optimised, with all the appropriate quality control protocols in place. Another hurdle is determining the best method of loading the drugs and quantification. Some methods such as sonication, though more efficient than other methods of drug loading such as incubation, cause damage to the structural integrity of the EVs [154]. Hence, the best method of loading drugs needs to be comprehensively evaluated. As the rapid clearance of EVs and hyperactivation of the immune system after administration are disadvantages of using EVs as drug delivery vehicles, the route of administration is crucial. [174]. Other drawbacks of using these drug delivery systems include the unavailability of a widely accepted production and purification process of EVs and the lack of reproducibility in drug loading techniques [178]. 

Taken together, extreme caution needs to be adhered to when using EVs in drug delivery. As one of the biggest reasons for using EV-based drug delivery is their ability to decrease cytotoxicity, drugs carrying EVs should demonstrate significantly better outcomes, tolerance, and safety than the existing cancer treatment. 

## 4. Conclusions and Future Directions

EVs form part of a group of small, heterogenous lipid nanoparticles that are released by virtually all cells in the body and participate in proximal and distal communication between cells [179]. They are also mediators of various types of physiological and pathophysiological processes. The majority of cell- and animal-based experimental evidence reviewed herein advocates the key role of EVs in almost every cancer hallmark characteristic ranging from cancer initiation to the various stages of cancer progression and paraneoplastic syndrome [180]. Although several pharmacological agents were developed to block tumour–EV biogenesis or secretion, none of them are clinically approved yet. Further studies with relevant pre-clinical models that recapitulate human cancer and clinical trials with the drugs that deplete tumour EVs might aid in the development of new anti-cancer therapies. 

Similar to LNP, EVs are phospholipid based; however, unlike LNPs, EVs are decorated with a complex range of surface proteins and lipids which aid in tissue homing. The composition of EVs and their biogenesis is directly contingent on their source. They can be derived from various cell types and sources including mammalian and bacterial cell cultures, bovine milk, blood plasma, and plants. Due to these characteristics and their natural nano-carrier qualities, EVs are being harnessed for the delivery of therapeutic payloads. Unlike artificially engineered nanoparticles, EVs are naturally occurring and hence non-inflammatory. The tissue-homing ability of EVs allows them to travel to distant target sites. There are several methods of loading drugs into EVs. Currently, various cancer types are treated with this novel strategy with promising outcomes and minimal to no side effects. In recent years, new methods of further improving these nanovesicles are being implemented, such as the development of EV-based super magnetic nanoparticles [164,181,182]. However, there are still many challenges that need to be addressed before commercializing drug-packed EVs for cancer treatment in humans. Therefore, further investigations are required to develop new strategies to improve the production of EVs and loading efficiency. 

## Data Availability

Not applicable.
